# A 2D Bismuth-Induced Honeycomb Surface Structure on GaAs(111)

**DOI:** 10.1021/acsnano.2c12863

**Published:** 2023-02-23

**Authors:** Yi Liu, Sandra Benter, Chin Shen Ong, Renan P. Maciel, Linnéa Björk, Austin Irish, Olle Eriksson, Anders Mikkelsen, Rainer Timm

**Affiliations:** † NanoLund and Department of Physics, Lund University, P.O. Box 118, 221 00 Lund, Sweden; ‡ Department of Physics and Astronomy, Uppsala University, P.O. Box 516, 751 20 Uppsala, Sweden; § School of Science and Technology, Örebro University, Fakultetsgatan 1, SE-70182 Örebro, Sweden

**Keywords:** bismuth, 2D layer, honeycomb structure, bismuthene, GaAs, STM, DFT

## Abstract

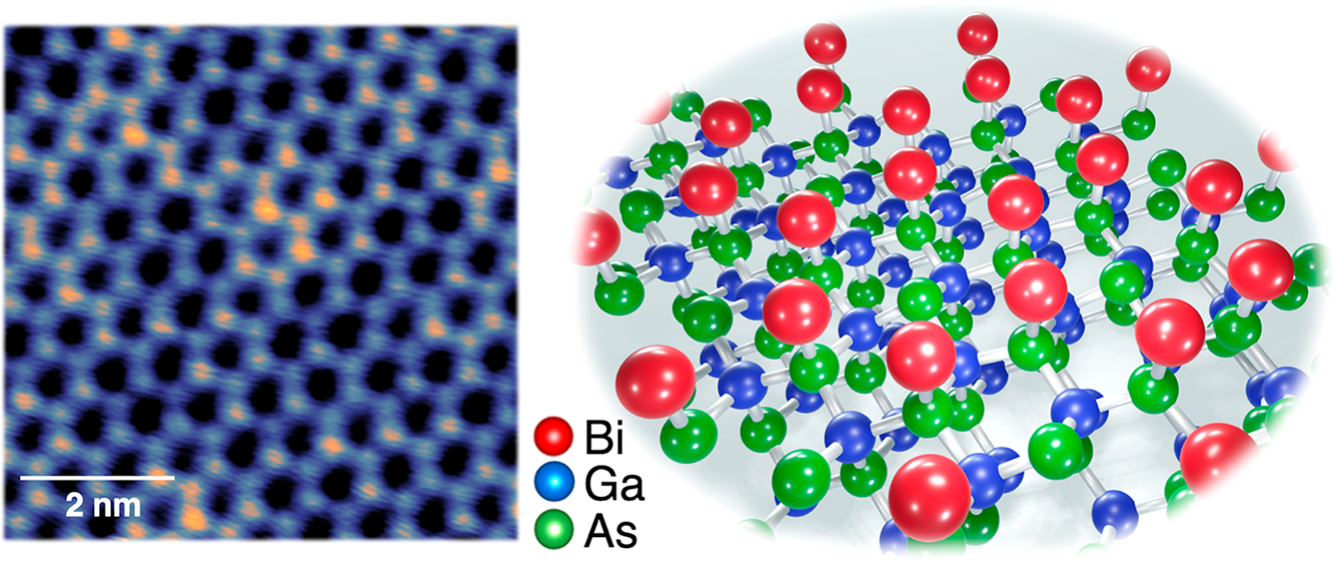

Two-dimensional (2D) topological insulators have fascinating physical properties which are promising for applications within spintronics. In order to realize spintronic devices working at room temperature, materials with a large nontrivial gap are needed. Bismuthene, a 2D layer of Bi atoms in a honeycomb structure, has recently attracted strong attention because of its record-large nontrivial gap, which is due to the strong spin–orbit coupling of Bi and the unusually strong interaction of the Bi atoms with the surface atoms of the substrate underneath. It would be a significant step forward to be able to form 2D materials with properties such as bismuthene on semiconductors such as GaAs, which has a band gap size relevant for electronics and a direct band gap for optical applications. Here, we present the successful formation of a 2D Bi honeycomb structure on GaAs, which fulfills these conditions. Bi atoms have been incorporated into a clean GaAs(111) surface, with As termination, based on Bi deposition under optimized growth conditions. Low-temperature scanning tunneling microscopy and spectroscopy (LT-STM/S) demonstrates a well-ordered large-scale honeycomb structure, consisting of Bi atoms in a √3 × √3 30° reconstruction on GaAs(111). X-ray photoelectron spectroscopy shows that the Bi atoms of the honeycomb structure only bond to the underlying As atoms. This is supported by calculations based on density functional theory that confirm the honeycomb structure with a large Bi–As binding energy and predict Bi-induced electronic bands within the GaAs band gap that open up a gap of nontrivial topological nature. STS results support the existence of Bi-induced states within the GaAs band gap. The GaAs:Bi honeycomb layer found here has a similar structure as previously published bismuthene on SiC or on Ag, though with a significantly larger lattice constant and only weak Bi–Bi bonding. It can therefore be considered as an extreme case of bismuthene, which is fundamentally interesting. Furthermore, it has the same exciting electronic properties, opening a large nontrivial gap, which is the requirement for room-temperature spintronic applications, and it is directly integrated in GaAs, a direct band gap semiconductor with a large range of (opto)electronic devices.

## Introduction

Two-dimensional (2D) materials with nontrivial topological properties are attracting high interest due to their fascinating physics and their large potential for spintronics and quantum technology. Quantum spin Hall (QSH) systems are such materials, which combine a bulk band gap with one-dimensional conductive edge states that are topologically protected, promising dissipationless spin current. Recently, bismuthene—a 2D, atomically thin layer of Bi atoms in a honeycomb structure—moved in the focus to realize QSH systems with an energy gap that is large enough for room temperature applications, mainly due to the large spin–orbit coupling of Bi. Reis et al. synthesized bismuthene on SiC^1^ and demonstrated a nontrivial energy gap of 0.8 eV, which is 6 orders of magnitude larger than that of graphene (0.8 μeV)^2^ and still 2 to 3 orders larger than those of silicene (2 meV)^3^ and germanene (24 meV).^3^ An even larger energy gap of about 1 eV was observed by Sun et al. for bismuthene on Ag(111).^4^ Generally, the size of the gap and even the existence of a nontrivial topological phase in bismuthene depend on the substrate material and its interaction with the Bi atoms.^4−6^ To combine spintronic applications enabled by bismuthene with conventional electronics or optoelectronics, it would be highly desirable to synthesize bismuthene directly on a semiconductor substrate with a direct band gap such as GaAs. Although Zhou et al. predicted the existence of bismuthene on Si with an energy gap of 0.8 eV already in 2014,^7^ bismuthene on a semiconducting substrate (with a band gap significantly smaller than that of SiC) has to our knowledge not been realized yet.

III–V semiconductors are especially promising for a wide range of (opto)electronic applications as they combine high charge carrier mobility with a direct band gap and offer great flexibility in combining different materials.^8^ GaAs is by now the most prominent III–V material. Thin films of Bi have successfully been grown on different III–V substrates.^9−12^ Continued Bi deposition typically leads to metallic Bi films or islands on top of e.g. InAs^9,10^ or GaAs,^11^ while the deposition of only about a monolayer of Bi can lead to Bi-induced reconstructions e.g. on GaAs(111),^13,14^ or even the otherwise nonreconstructed InAs(110) surface.^15^ Bi-induced surface reconstructions have also been observed on (001) surfaces of MBE-grown diluted GaAsBi films.^16,17^ McGinley et al. investigated the Ga-terminated (111) surface,^14^ called (111)A, and the As-terminated (111) surface,^13^ called (111)B, of GaAs upon deposition of about 3 Å of Bi and subsequent annealing and observed with X-ray photoelectron spectroscopy (XPS) the existence of Ga–Bi and As–Bi bonds, respectively, in addition to bulk Ga–As and metallic Bi–Bi bonds. Bi–Bi bonds and Bi–As bonds have also been predicted by density functional theory (DFT)^18,19^ and observed by XPS^18^ due to Bi dimers and mixed Bi–As dimers forming the reconstructed surface of GaAs:Bi(001). These results confirm that it is possible to obtain a high density of Bi atoms at least locally on III–V surfaces, with Bi forming bonds to the III–V substrate. Chuang et al. predicted, based on first-principles calculations, that the binary combination of group-III and Bi atoms can form 2D topological insulators.^20^ The strength of the band inversion would depend on the type of group-III element, where the calculations for GaBi result in a direct inverted band gap of 0.19 eV at the Γ point. However, pure GaBi compounds or layers have not been found until very recently, when we observed GaBi islands on the {112̅0} surfaces of wurtzite GaAs, which only exists as a stable crystal phase in GaAs nanowires.^21^ Yielding a stable, large-scale well-ordered GaAs:Bi structure, where Bi atoms are incorporated in the group-V lattice positions, involving the distinct Bi-introduced electronic states, remains a highly desirable but yet unresolved target.

Here, we present a well-ordered 2D Bi-induced honeycomb structure which is formed after Bi deposition at 250 °C on GaAs(111)B as observed by atomically resolved low-temperature scanning tunneling microscopy (LT-STM). An almost defect-free, large-scale honeycomb structure is obtained after short anneal. The GaAs(111)B template consists of bilayers of Ga and As atoms, with an As-terminated surface. By a systematic study of deposition and annealing steps we can understand the Bi incorporation process. Bi–As bonds, indicating the successful Bi incorporation, are observed by XPS after Bi deposition on a sample at 250 °C, while metallic Bi–Bi bonds are dominant after extended room temperature Bi deposition. Based on the experimental findings, a model is established by density functional theory (DFT) of the fully flat 2D surface crystal structure, which can be described as a Bi √3 × √3 30° overlayer on GaAs(111)B. The lattice constant of the honeycomb structure is 0.69 nm, which is significantly larger than those of previously observed bismuthene on SiC with 0.535 nm^1^ and bismuthene on Ag(111) with 0.57 nm.^4^ Furthermore, the honeycomb structure observed here consists of Bi atoms which are covalently bonded to As atoms underneath but do not form strong bonds between each other. This bonding configuration is in contrast to previously observed bismuthene flakes, which showed strong metallic Bi–Bi bonds in XPS,^5^ and to Xene layers such as Graphene in general, but it might be explained by the larger distance between neighboring Bi atoms in the honeycomb structure studied here. Nevertheless, the DFT model of this GaAs:Bi honeycomb structure results in an electronic band structure with a nontrivial gap at the K point, analogous to that of previously observed bismuthene.^1,4^ The calculations are supported by scanning tunneling spectroscopy (STS) measurements, which show Bi-induced states within the GaAs band gap. Accordingly, the GaAs:Bi honeycomb structure can be understood as a structurally different form of bismuthene, which combines fresh fundamental insight into the important class of 2D Xene structures with the promise for future spintronic applications.

## Results and Discussion

**Figure 1:**
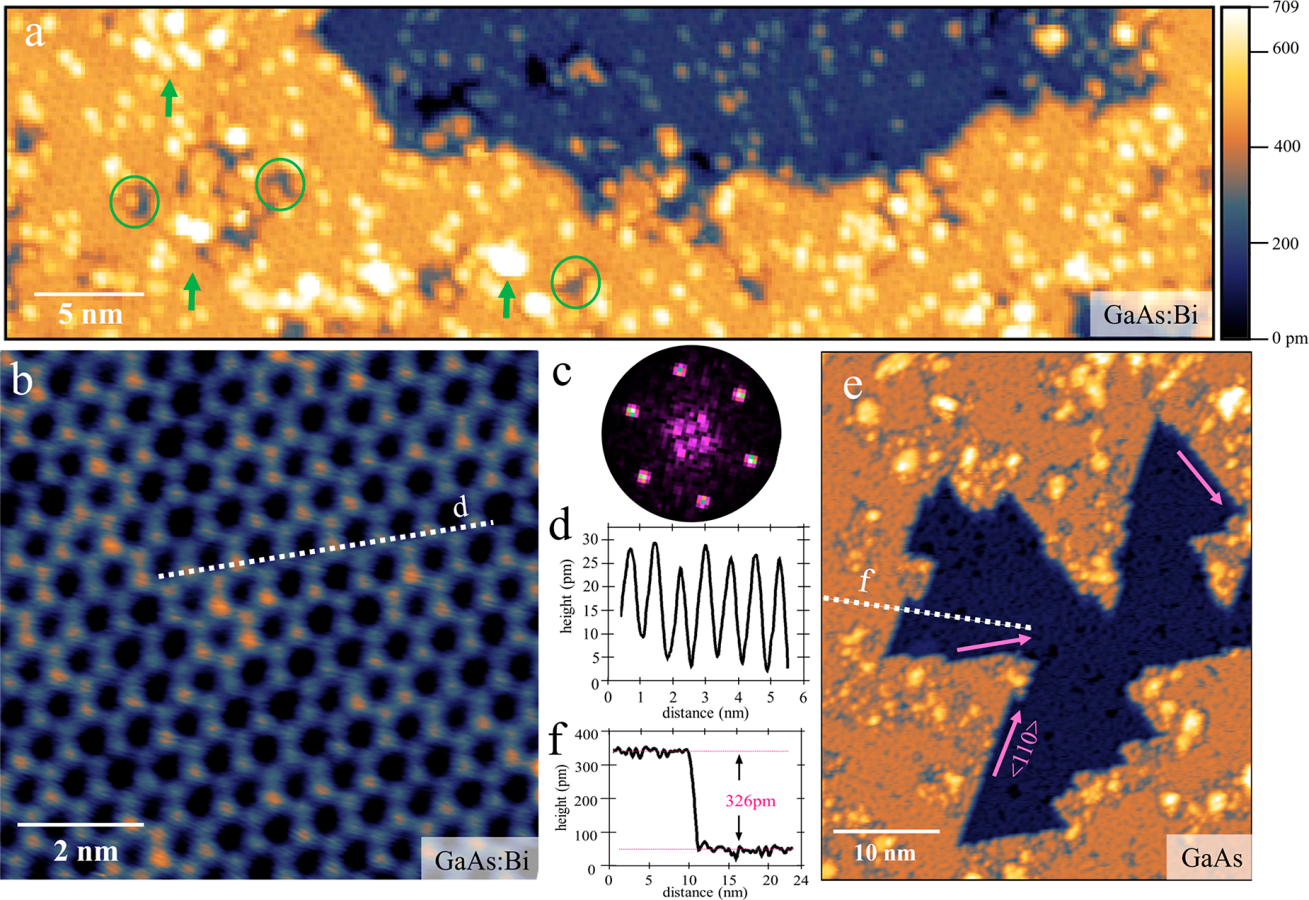
Honeycomb structure upon Bi deposition on GaAs(111)B at 250 °C. (a) Overview STM image of the honeycomb structure. Green circles indicate some hollow areas and green arrows some Bi clusters on the surface. (b) Atomically resolved STM image of the honeycomb structure. Each bright dot presents an individual Bi atom. The STM color scale extends over 44 pm. (c) Fast Fourier transform (FFT) image of image (b), showing a clear single periodicity of sixfold symmetry. The additional weak spots close to the center are probably related to surface step edges of the underlying GaAs(111)B substrate. (d) Height profile of a line scan across the honeycomb structure as indicated in (b) by a white line. (e) Overview STM image of the clean GaAs(111)B surface, for comparison. Pink arrows indicate step edges of the surface terraces along ⟨110⟩ directions. (f) Height profile of the clean GaAs surface, as indicated by the white line in (e), showing the surface step height. STM imaging parameters are *V*_T_ = −3 V, *I*_T_ = 50 pA for (a), *V*_T_ = −5 V, *I*_T_ = 100 pA for (b) and *V*_T_ = −3 V, *I*_T_ = 80 pA for (e).

Initially, a clean GaAs(111)B surface was obtained by annealing a GaAs substrate in the presence of atomic hydrogen, provided by a thermal cracker. This treatment has been shown before to efficiently remove native oxides from GaAs^22^ and other III–V surfaces.^23^ LT-STM images of the clean surface, as the one presented in Figure 1e, show large, triangular-shaped, atomically flat terraces with step edges along ⟨110⟩ directions, typical for (111) surfaces. Most of these step edges have a height of about 0.33 nm, as shown in Figure 1f, which corresponds to a monolayer of GaAs in the (111) plane. More details about the clean GaAs surface can be found in the Supporting Information (SI), especially in Figure S1.

### Bi-Induced Large Scale Honeycomb Structure on GaAs(111)B

Upon Bi deposition on the as-cleaned GaAs(111)B substrate at 250 °C, a full layer of uniform honeycomb-like networks is formed. Overview and atomically resolved STM images of the honeycomb structure are shown in Figure 1a and 1b, respectively. The ordered honeycomb network is extending to a full monolayer but including some small gaps, seen as hollow areas. In addition, clusters of excessive Bi, with the size of a few nm, are found on the surface. The sixfold symmetry of the honeycomb structure is clearly confirmed by fast Fourier transformation (FFT) patterns as the one shown in Figure 1c. The six diffraction points look sharp without signs of interference, indicating that no Moiré patterns form between the honeycomb structure and any other periodic layer underneath. The flatness of the honeycomb structure is measured by tracking the apparent height in the STM images, with height profiles shown in Figure 1d. A height fluctuation of only around 22 pm confirms that this honeycomb network is very uniformly distributed and atomically flat. Moreover, the lattice constant of the honeycomb structure can be acquired by measuring lateral distances in these height profiles, amounting to a periodicity of 0.75 ± 0.04 nm and an atomic nearest neighbor distance of 0.44 ± 0.06 nm. The appearance of the honeycomb structure as shown in Figure 1a is not limited to individual flakes, but extends over the entire mm-scale substrate.

**Figure 2:**
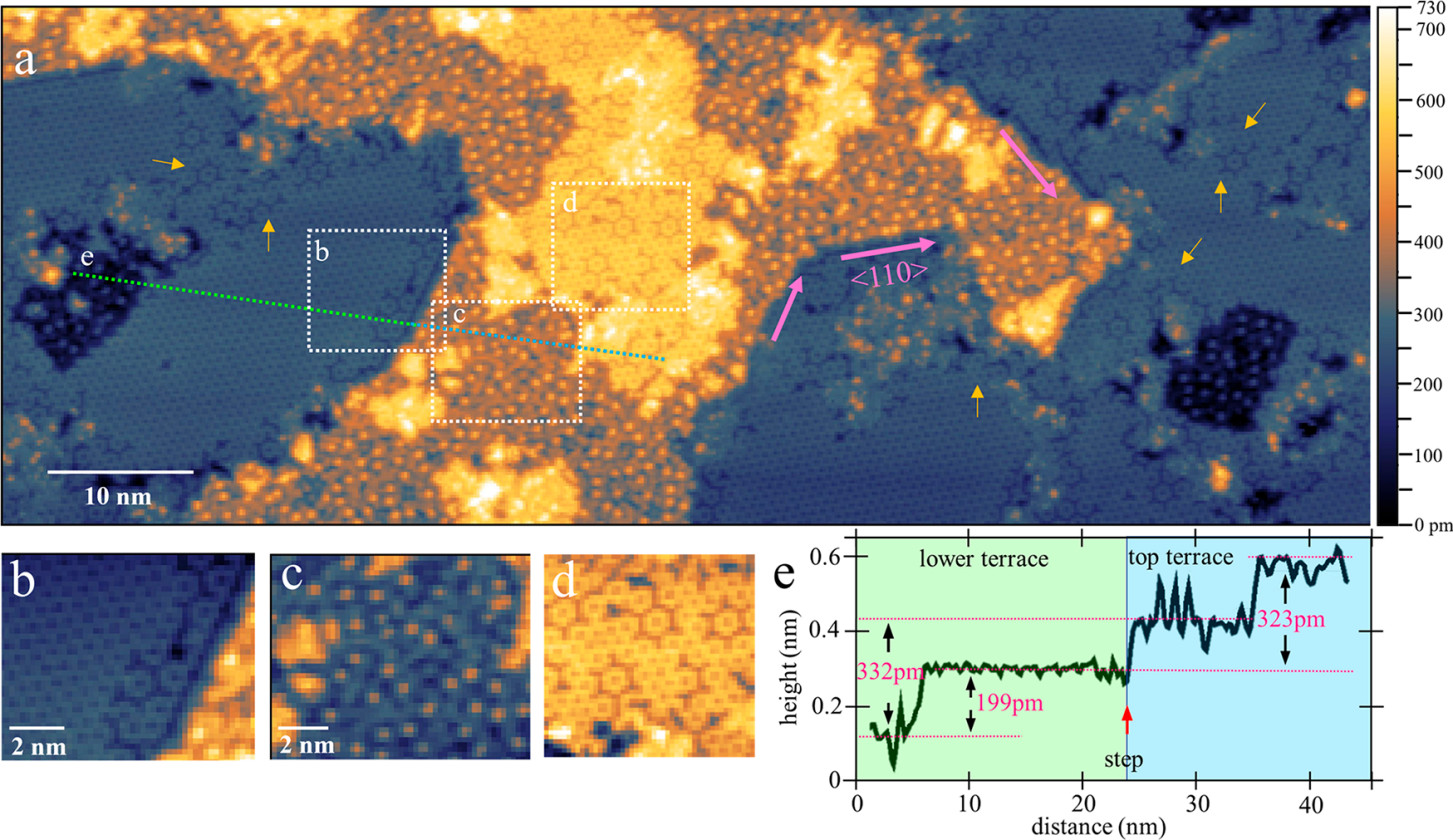
Bi-induced honeycomb structure on GaAs(111)B after quick annealing at 400 °C. (a) Overview STM image. Yellow (blue) zone presents the top (lower) surface terrace. Some step edges along ⟨110⟩ orientations are marked with pink arrows; yellow arrows point out areas with an incomplete honeycomb structure. (b,c,d) Close-view STM images of the selected areas marked in (a), presenting different topographies. (e) Line scan along the green dashed line in (a), showing the height profile of the lower terrace (background in green) and the top terrace (background in blue). STM color scale extends over 421, 522, and 316 pm in (b), (c), and (d), respectively. *V*_T_ = −3.5 V, *I*_T_ = 50 pA for all STM images.

After annealing at around 400 °C for 10 min, two different types of patterns dominate the surface, as can be seen in the STM overview image of Figure 2a. There are large terraces of almost perfect honeycomb structure, without any Bi clusters left on the surface. Between these honeycomb terraces, areas with a more irregular, dotty appearance are found (below called dotted layer). Still, all honeycomb domains show the same lattice orientation as before. With clear step edges along ⟨110⟩ directions (marked with pink arrows in Figure 2a) on GaAs(111)B, it can be concluded that the honeycomb structure follows the lattice orientation of the GaAs(111)B substrate, with a missing atom in the center of each honeycomb, thus forming a √3 × √3 30° reconstruction. While most of the honeycomb domains show a perfect, regular surface coverage (see Figure 2a and left part of Figure 2b), there also exist areas of an incomplete honeycomb structure, where missing atoms form a nm-scale periodic pattern, as shown in Figure 2d and marked by yellow arrows in Figure 2a. Furthermore, most honeycomb domains have atomic vacancies close to their boundaries, where also individual hexagons at irregular positions can be found, as shown in the right part of Figure 2b.

The domains of the dotted layer, as shown in Figure 2c, are characterized by a high density of irregularly distributed, atom-like bright dots surrounded by a nm-scale pattern of slightly varying contrast. The brightness of these dots in the STM images is comparable to that of the individual atoms in the honeycomb structure, indicating that both features might be due to Bi atoms on the top surface layer of the sample. To further investigate the relation between the honeycomb structure and the dotted layer, a line scan was taken along honeycomb and dotted layer domains of neighboring terraces, as indicated in Figure 2a, with the corresponding height profile shown in Figure 2e. The height profile presents two types of areas in both the lower terrace (background in green) and top terrace (background in blue). We can see that on both terraces, the honeycomb structure (referring to the bright atoms in the honeycomb mesh) is about 200 pm higher than the bottom of the dotted layer (referring to the darker pattern of this layer, not the individual bright dots). This height difference is less than a single atomic layer of the GaAs(111) surface, which amounts to 326 pm, but is in a range which can be expected for the height of individual Bi atoms on top of a GaAs structure. Furthermore, the height difference between adjacent dotted layers (or adjacent honeycomb structures) is around 0.33 nm. We should point out that the STM height profiles of Figure 2 are taken at a high tunneling bias of −3.5 V, at which we can expect the apparent height to depend mainly on topography and only weakly on electronic effects. By considering these height differences and the appearance of the domains, we interpretate the dotted layer structure to be the GaAs(111)B surface with individual, scattered Bi atoms with a density of about 1 atom per nm^2^ on top, while the honeycomb structure is based on the same GaAs(111)B terrace, but with a higher Bi atom density resulting in the regular honeycomb structure (or in the incomplete honeycomb structure with a periodic pattern of vacancies, if the Bi atom density of the complete honeycomb structure is not reached). The assumption that the honeycomb structure is formed by Bi atoms will be confirmed by XPS results discussed below.

According to this interpretation of the surface structure, step edges of the dotted layers are determined by the surface terrace structure of the GaAs(111)B surface, while the sizes of the honeycomb domains within one terrace (surrounded by the dotted layer structure) are due to the total amount of Bi atoms on the surface. By evaluating many STM images like the one shown in Figure 2a, we obtain an average surface coverage with Bi atoms of about 38%, compared to the amount of Ga or As lattice sites. Upon continued annealing at 400 °C, the coverage of the (111)B surface with honeycomb domains is further reduced, down to zero coverage. At this point, the surface looks similar to the clean GaAs(111)B substrate, which indicates the desorption of Bi atoms from the GaAs(111)B surface during annealing.

### Bi–As and Bi–Bi Bonds Observed by X-ray Photoelectron Spectroscopy

To further investigate the bonding configuration and chemical state of Bi atoms, synchrotron-based XPS has been used to characterize the sample upon Bi deposition and at following processing steps. A photon energy of 120 eV was used, corresponding to very surface-sensitive conditions. Samples were prepared by deposition of Bi onto clean GaAs(111)B at a temperature of 250 °C, followed by 10 min of annealing to 400 °C, in the same way as for the STM study. This initial process, which according to the STM images results in a well-ordered honeycomb layer of Bi atoms, is in the following referred to as Bi incorporation (“Bi incorp.”)—the physical meaning of this term will become apparent in the discussion of the XPS results. To identify and distinguish Bi atoms in different chemical states and bonding environments, two additional Bi deposition steps at room temperature (below referred to as “Bi_RT_1” and “Bi_RT_2”) and two subsequent thermal annealing steps at 250 and 400 °C (below referred to as “Anneal_1” and “Anneal_2”) were added after the initial Bi incorporation process.

**Figure 3:**
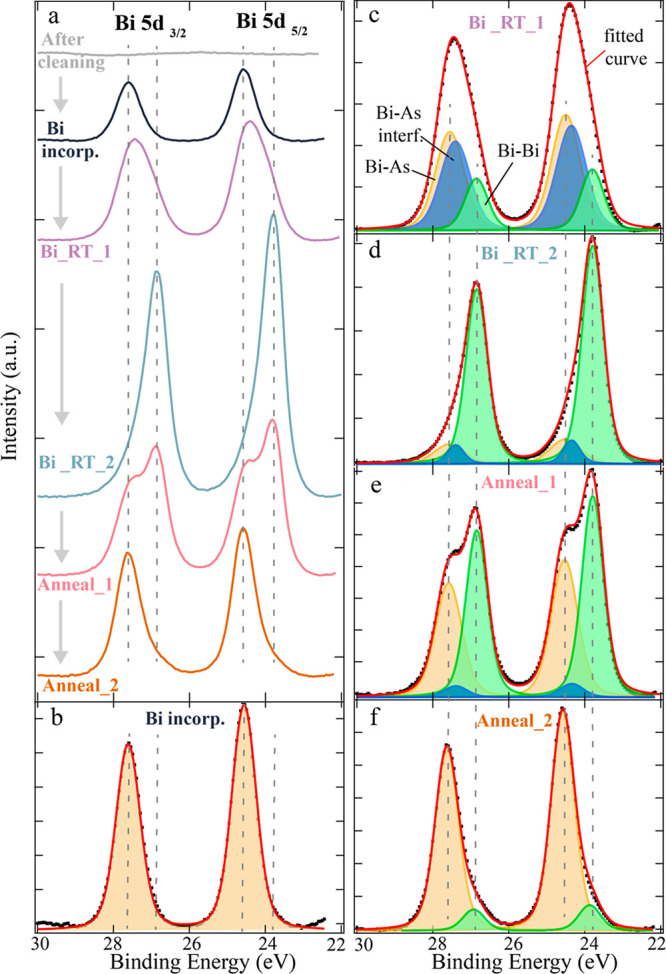
XPS Bi 5d core level spectra at after subsequent processing steps. (a) Overview of Bi 5d spectra along the sample processing timeline (from top to bottom), as described in the main text, after background subtraction. (b–f) Component decomposition and curve fitting of the spectra shown in (a). Corresponding components are marked in (c). The evolution of the Bi intensity upon subsequent processing steps can be followed in (a), where the spectra are shifted along the intensity axis for better visualization, while the intensities are normalized in (b)–(f). Dashed lines in all figures indicate the binding energy (B.E.) position of Bi–As (24.5 and 27.6 eV) and Bi–Bi (23.8 and 26.9 eV) for both 5d_3/2_ and 5d_5/2_ core levels.

XPS spectra of the Bi *5d* core level obtained after each preparation step (from top to bottom) are shown in Figure 3a. Only one doublet with a binding energy (B.E.) of the Bi *5d*_*5/2*_ peak of 24.5 eV is needed to fit the spectrum after the initial Bi incorporation process, as can be seen in Figure 3b, indicating that at this stage all Bi atoms on the surface are in the same chemical state. After two subsequent Bi deposition steps at room temperature, another component with a 0.72 eV smaller B.E. is dominating the spectrum, as shown in Figure 3d. By comparing the absolute intensities of the spectra “Bi incorp.” And “Bi_RT_2” in Figure 3a, it becomes evident that a significantly larger amount of Bi can be found on the sample after extended room temperature deposition. This thicker Bi layer grown at room temperature should consist of metallic Bi, according to previous reports;^9,11^ thus we attribute the peak at lower B.E. to metallic Bi. The component at higher B.E. is then assumed to be Bi bonded to As, in agreement with conclusions from literature.^13,24^

These XPS results indicate that, after initial Bi deposition at increased sample temperature and a short annealing, all Bi surface atoms are in the same chemical environment, characterized by Bi–As bonds. This is consistent with the STM results from the corresponding surface (see Figure 2) that all single Bi atoms have similar heights and, thus, are very likely to be in the same bonding configuration. When additional Bi is deposited onto the sample at room temperature (see Figure 3c and 3d after step Bi_RT_1 and Bi_RT_2, respectively), the majority of these Bi atoms form Bi–Bi bonds, indicating growth of metallic Bi islands or layers. However, this metallic Bi is not stable at higher temperatures: Upon annealing to 250 °C, the total amount of Bi and especially the Bi–Bi component decreases significantly (see Figure 3a and 3e), and after annealing to 400 °C, the Bi–Bi component is almost completely removed, as shown in Figure 3f. This is also consistent with previous studies showing that metallic Bi can be fully desorbed at around 350 °C.^14^ It is important to note, though, that a strong Bi–As component is found in the XPS results even after annealing to 400 °C, confirming that these bonds are more stable. Overall, the XPS results together with the STM images show that the Bi atoms in the honeycomb structure are not physically absorbed on the surface, but form a stable, chemical bond to the As atoms on the GaAs(111)B substrate. Since we only see one component in the XPS data after the initial Bi incorporation process, we conclude that even the Bi atoms of the dotted structure seen by STM have formed bonds to As atoms underneath, even though the density of the Bi atoms is not sufficient to form a honeycomb domain. This agrees with the apparent height of the Bi atoms in the STM images which is the same for the dotted domain and the honeycomb structure. Interestingly, no sign of Bi–Bi bonds is found by XPS after the initial Bi incorporation process, which corresponds to the observation of the honeycomb structure in STM. Accordingly, the Bi atoms in this structure are only bonded to the As atoms underneath, but do not have covalent bonds between each other. This is in contrast to a previously observed bismuthene structure,^5^ where the XPS signal from the Bi *4f* core level was reported to be dominated by metallic Bi.

Beside the Bi–As and Bi–Bi bonds, another component is needed to successfully fit the Bi *5d* spectra after the first Bi deposition at room temperature (step Bi_RT_1), colored in blue in Figure 3c. The intensity of this component drops substantially upon annealing at 250 °C (Figure 3e), and it is completely removed after annealing to 400 °C (Figure 3f). A possible explanation of this component can be that as Bi is a metallic material, the additionally deposited Bi adatoms at room temperature, which form Bi–Bi bonds to the Bi surface atoms deposited previously, weaken the polar Bi–As bond of those Bi surface atoms, therefore shifting the Bi–As peak of the Bi *5d* core level to lower B.E. by about 0.2 eV. A similar chemical shift due to extra surface metal deposition has also been observed in other material systems.^25^ For Bi atoms deposited on GaAs(111)B, the chemical shift between Bi *5d* Bi–Bi and Bi–As components has been reported to increase from about 0.5 eV at room temperature to about 0.7 eV at elevated temperatures^13^—this observation might be due to the same phenomenon that we observe here. After the Bi_RT_1 deposition step, the previously topmost Bi surface atoms have become the interface layer between GaAs(111)B and the layer of metallic Bi; thus, we refer to the component of Bi–As with slightly lower B.E. as the Bi–As interface (“Bi–As interf.”) in Figure 3. It is worth pointing out that the Bi–As interface peak has a similar intensity as the original Bi–As component in both Figure 3c and 3d, indicating that half of the interface Bi atoms with Bi–As bonds also have Bi–Bi bonds to atoms of the metallic Bi layer on top. The strong decrease of the Bi–As interface component upon annealing goes hand in hand with the desorption of the metallic Bi layer, visible by a decrease of the Bi–Bi component. This confirms the assumption that the presence of metallic Bi–Bi bonds is responsible for the small shift of the Bi–As B.E.

While strong changes can be observed in the Bi *5d* core levels upon subsequent Bi deposition and annealing steps, only small changes can be seen in the As *3d* core level spectra, which are shown in Figure S3a of the Supporting Information. As–Ga and As–Bi bonds are expected to have very similar B.E.s and thus cannot easily be distinguished.^14^ However, a small shift by about 0.1 eV of the entire As *3d* spectrum toward higher B.E. is observed after deposition step Bi_RT_1. This shift might partly be explained by the same mechanism that creates the Bi–As interface component in the Bi *5d* spectra, as a less ionic character of the Bi–As bond will lead to a shift toward the higher B.E. of the corresponding As *3d* component. Furthermore, upon the same deposition step Bi_RT_1 also the Ga *3d* core levels slightly shift toward higher B.E., though by less than 0.1 eV, as shown in Figure S3b of the Supporting Information. A shift of both the Ga *3d* and As *3d* core levels toward higher B.E. might indicate a shift of the Fermi level within the GaAs band gap, toward the conduction band, i.e. making the GaAs less *p-*type, when a metallic Bi layer is deposited on top of the GaAs substrate. Otherwise, no significant changes of the Ga *3d* core-level spectra can be observed. According to literature,^14^ Ga–Bi bonds should result in a peak at 0.3 eV lower B.E. than that of Ga–As; thus, the presence of a significant amount of GaBi can be excluded.

Combining STM and XPS results, we can conclude that Bi deposition on a heated GaAs(111)B surface results in the topmost As atoms bonding with Bi atoms, forming Bi–As covalent bonds. These Bi–As bonds and the corresponding Bi–As layer are qualitatively different from metallic Bi islands or Bi trimers in Bi-induced surface reconstructions which have been seen before upon Bi deposition on GaAs or other III–V semiconductors.^9,13,16,18^ Here, a large-scale, ordered 2D honeycomb structure is formed by Bi–As bonds, which even is stable upon annealing up to 400 °C.

### Electronic Contrast of Bi Honeycomb Structure

**Figure 4:**
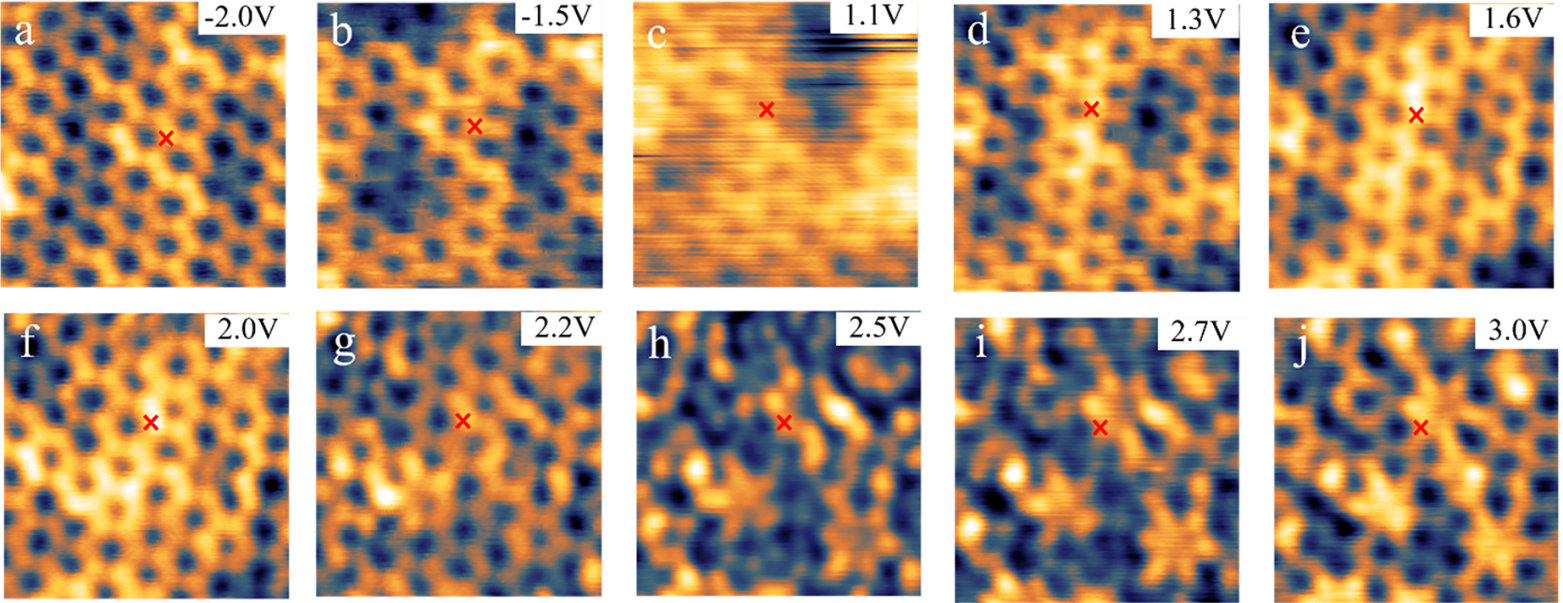
LT-STM images of the Bi-induced honeycomb structure under different tunneling bias, representing different LDOS distribution. (a–j) The same area of a Bi-induced honeycomb structure with tunneling bias ranging from −2.0 V to +3.0 V. The red cross in each image marks the same physical position. All STM images are taken at around 10K. The STM color scale extends over 1.0, 0.9, 2.0, 1.2, 0.9, 0.9, 1.1, 1.0, 0.9, and 0.7 pm in (a)–(j), respectively.

In STM images, As (Ga) atoms can be observed under negative (positive) bias due to the charge polarity nature in an As–Ga bond. Surface Bi atoms, on the other hand, can show either filled valence band (VB) states under negative bias or empty conduction band (CB) states under positive bias, depending on their bonding configuration. Comparison of STM images under different bias thus gives additional information about which type of atoms are present in the honeycomb structure. Furthermore, the spatial distribution of filled and empty electronic states can be seen. A chosen set of LT-STM images under sample voltages ranging from −2.0 V to +3.0 V is shown in Figure 4. For easy comparison, the red cross marks the same coordinates in each image, the slight change of the position of the red marks is due to the small thermal shift during STM scanning. Tunneling conditions are unstable in a bias range from −1.4 V to +1.0 V due to the existing band gap; thus, no images can be acquired at these voltages.

All STM images of Figure 4, independent of the bias, show an overlay of the honeycomb structure with almost isotropic, constant DOS located at the individual atoms, superimposed with an unordered electronic contrast showing some irregular nm-scale patterns. Only at high positive bias these irregular patterns have a stronger influence on the overall contrast in the STM images, while at a bias of less than 2.5 V at both polarities the honeycomb structure is dominating. At a larger negative bias, which was chosen in the STM images of Figure 2, the same type of irregular, nm-scale contrast pattern was only seen as a background in the images of the dotted layer. We attribute this pattern to arise from local fluctuations of the DOS of the GaAs substrate, likely due to the distribution of dopants or defects, which here is overlaying with the LDOS of the honeycomb layer. Importantly, one can clearly see that the same atomic positions of the honeycomb structure appear bright at both negtive and positve bias. Since Ga and As atoms cannot be observed under both bias polarities, this is another indication that the honeycomb structure consists of a complete layer of Bi atoms.

### First-Principles Atomistic Modeling of the Bi Honeycomb Structure

**Figure 5:**
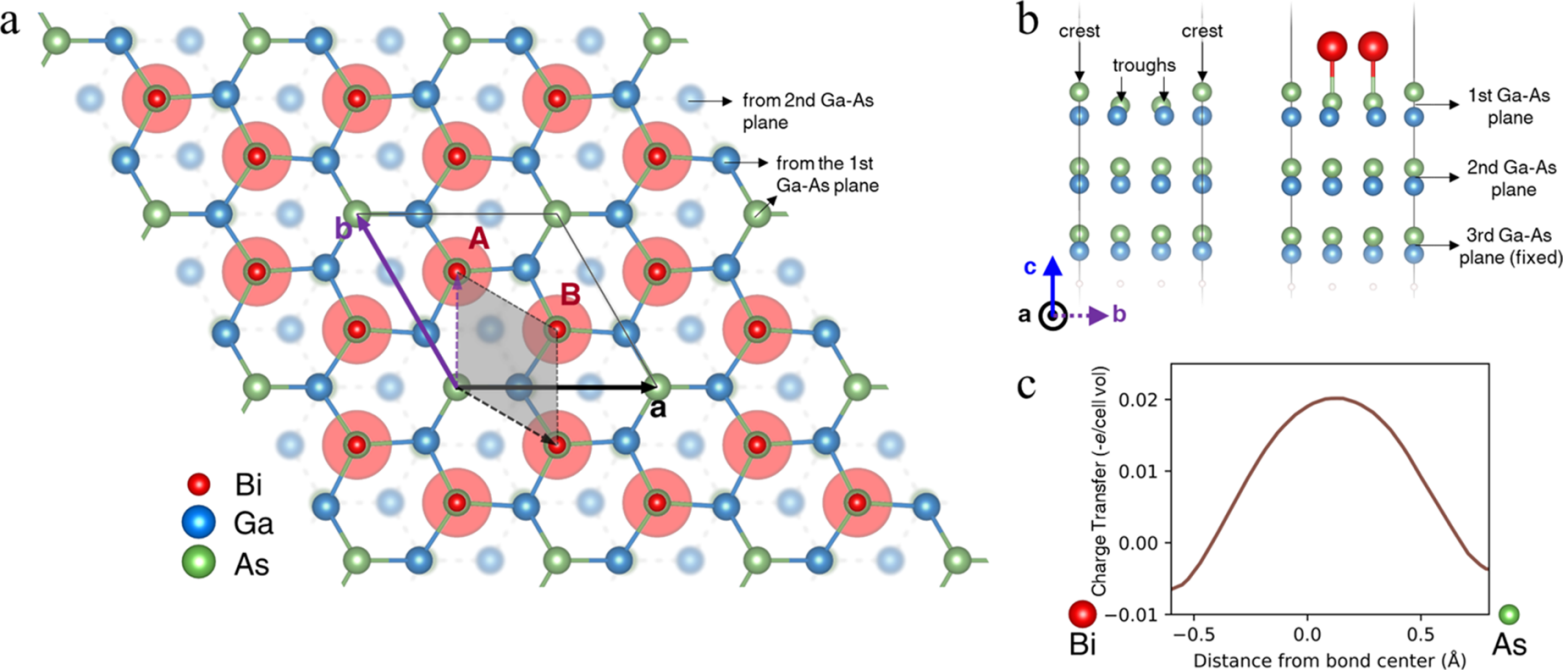
First-principles atomistic model of the Bi honeycomb structure. (a)Top view of the Bi-induced honeycomb structure on top of the GaAs(111)B substrate. The positions of the Bi atoms are emphasized with shaded red circles to highlight its honeycomb lattice that is made up of two sublattices, A and B. The hollow site is set as the origin of the unit cell of the honeycomb lattice structure and **a** and **b** define the lattice vectors of the absorbate unit cell. The substrate unit cell is also indicated, shaded in gray and defined using dashed lattice vectors. We see that the absorbate unit is rotated 30° anticlockwise with respect to the substrate and that each absorbate lattice vector is √3 times the length of the corresponding substrate lattice vector, i.e., Bi forms a√3 × √3 30° overlayer in the Wood’s notation. (b) Side view of the GaAs(111)B substrate with (right) and without (left) the honeycomb lattice. The first two Ga–As planes of the substrates are fully relaxed by minimizing the total energy of the system while the third plane is fixed in position to model the bulk. The faint atoms at the bottom of the slab are the pseudohydrogen atoms used to saturate the bonds. On the left part of (b), one sees that surface reconstruction of the pristine GaAs(111)B surface results in crests and troughs at the surface. When deposited, Bi preferentially occupies the troughs, forming Bi–As bonds with the substrate (as shown), and the crests become the hollow sites of the honeycomb lattice (right part of (b)). (c) Calculated charge transfer plot in units of electron charge per supercell volume, along a Bi–As bond. The plot shows a strong localization of charge transfer at the Bi–As bond center.

The atomic arrangement of the Bi-induced honeycomb structure on top of the GaAs(111)B substrate was modeled using DFT calculations. In its most stable configuration, the Bi atoms are located directly on top of the As atoms from the top Ga–As plane. From Figure 5a, it is clear that the periodic Bi honeycomb monolayer follows the symmetry of the underlying GaAs(111)B surface. The center of each hexagon is a hollow site, at which an As atom is not bonded to a Bi atom above, representing a Bi coverage of 67% of all As sites. Therefore, the surface unit cell of the honeycomb layer is larger than the primitive unit cell of the GaAs(111) substrate, corresponding to the periodicity of a √3 × √3 30° overlayer in the Wood’s notation. The calculated distance between the nearest hollow sites, i.e., the lattice constant of the honeycomb structure, is 0.69 nm, which is slightly shorter than the experimental value of 0.75 ± 0.04 nm. Similarly, the distance between two nearest Bi atoms is calculated to be 0.40 nm, agreeing very well with the experimentally measured distance of 0.44 ± 0.06 nm. After relaxation, all Bi atoms in the calculations have the same height. Thus, the small (unordered) fluctuations observed experimentally (see Figure 4 and Figure S2 of the Supporting Information) are not due to the honeycomb structure itself, but arise from the LDOS of the GaAs(111)B substrate probably due to the nm-scale dopant (or defect) distribution in the subsurface layers.

From a surface energy perspective, the calculations show that, upon Bi deposition, it is energetically most favorable for the Bi atom to sit directly above the As atom of the (As-terminated) GaAs(111)B surface (Figure 5a and 5b). When the (111)B surface was relaxed in the absence of Bi deposited (Figure 5b, left), the surface reconstruction results in periodic topographical modulation of the crystal surface in the form of crests and troughs (with heights varying with ±0.33 Å). When Bi is deposited, it preferentially occupies the troughs before being incorporated by forming Bi–As bonds (Figure 5b, right). The crests remain preferentially unoccupied and become the hollow sites of the honeycomb lattice. The formation of the Bi–As bonds has a negative formation energy that is calculated (see the Methods section) to be −0.81 eV per unit cell of the honeycomb lattice (Figure 5a). This strongly exothermic bond formation explains the stability of the Bi–As bonds and the honeycomb structure which was observed experimentally even upon annealing. This is also in line with the expectation that Bi, like Ga, has the oxidation state of 3, and therefore, Bi–As bonds, like the Ga–As bonds, should be more favorably formed than Bi–Ga bonds. In the DFT calculations, when Bi atoms were instead placed directly above the Ga atoms from the first Ga–As plane or the Ga atoms from the second Ga–As plane and only allowed to relax in the out-of-plane direction, the formation energies were in both cases found to be 0.30 eV per honeycomb unit cell, confirming that these structures would be much less favorable. We also considered the case of Bi substituting As, i.e., Bi_As_, at a coverage of 11% (one Bi per honeycomb unit cell), and found that a formation energy of −0.16 eV per honeycomb unit cell would be needed, making this configuration less likely to form experimentally than the honeycomb lattice structure.

Next, the charge transfer obtained from DFT was evaluated along several possible bond directions. As Figure 5c shows, there is a strong localization of charge transfer between the Bi and As atoms, which is consistent with covalent bond formation. Here, charge transfer is defined as the charge density minus the superposition of atomic densities. However, only very weak interactions exist between Bi–Bi and Ga–Bi pairs. These results are also consistent with our XPS data, which shows that only the Bi–As bonding configuration is present after Bi deposition and incorporation (Figure 3b), while the Bi–Bi bonds only show up after Bi deposition at room temperature. This is in contrast to reported XPS studies of free-standing bismuthene,^5^ where the Bi *4f* core-level spectra were dominated by metallic Bi–Bi bonds. We relate this different bonding configuration to different properties of the atomic structure: Combined STM and DFT studies have been published for bismuthene on SiC^1^ and for bismuthene on Ag(111),^4^ with lattice constants of the bismuthene honeycomb structure amounting to 0.535 and 0.57 nm, respectively. Our model results in a lattice constant of the GaAs:Bi honeycomb structure of 0.69 nm. The more than 20% larger distance between neighboring Bi atoms makes Bi–Bi bonds less favorable.

**Figure 6:**
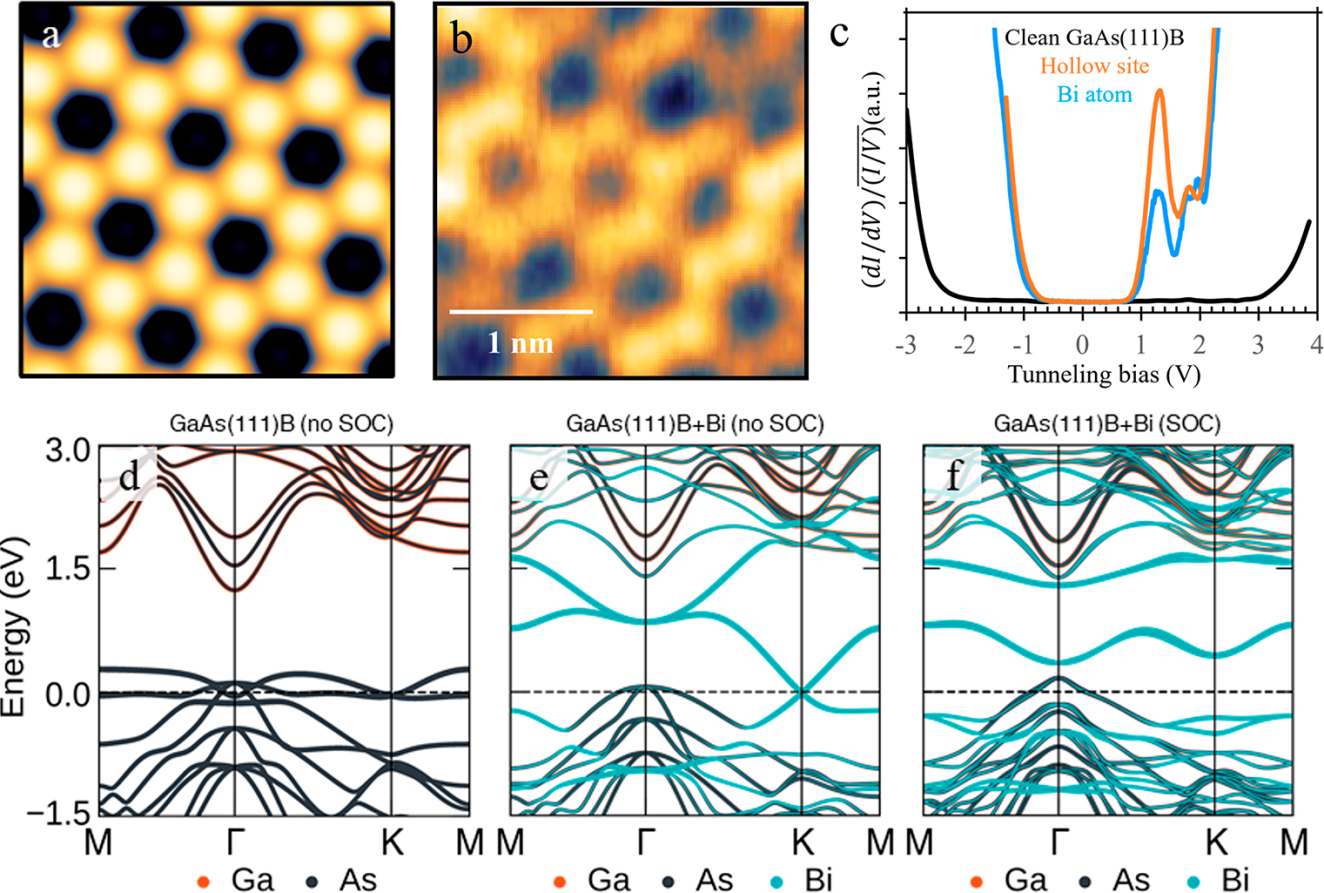
Electronic properties of the honeycomb structure as-deposited on the GaAs(111)B surface. (a) Calculated STM image using DFT based on the Tersoff–Hamann model. (b) Experimental STM image, acquired at *V*_T_ = −2.0 V, *I*_T_ = 350 pA. (c) LT-STS () point spectra, obtained at a Bi atom (blue) and a hollow site (orange) of figure (b) and at a clean GaAs(111)B surface (black). (d–f) DFT band structures of the GaAs(111)B surface with and without Bi. The band states are projected onto the atomic pseudowave function of Ga 4p and 5s orbitals (orange), As 4p and 5s orbitals (black), and Bi 6p_*x*_ and 6p_*y*_ orbitals (teal), with the thickness of the line representing the degree of projection. The Fermi level is at 0 eV. (d) DFT band structure of the pristine (111)B surface of a GaAs substrate with no Bi. (e) DFT band structure of the honeycomb structure as-deposited on the GaAs(111)B surface, calculated without spin–orbit coupling (SOC). There are two overlapping Dirac cones at the K point. (f) DFT band structure of (e) calculated with SOC. SOC leads to the opening of a nontrivial gap at the Dirac cones, and the splitting of the double degenerate bands in the low-energy region near the Fermi level.

Using the Tersoff–Hamann model,^26^ we further simulated an STM image of the honeycomb structure using the DFT wave functions under the constant-current mode. The calculated STM image, shown in Figure 6a, agrees very well with the experimental STM image of Figure 6b. Concluding this section, under the optimized incorporation conditions, the deposited Bi occupies the troughs on the GaAs(111)B surface, before overcoming the energy barrier needed for the Bi–As bond formation to be incorporated, resulting in the lowest-energy configuration of the ordered honeycomb pattern.

### Surface Electronic Properties of the Honeycomb Structure

Based on the structural model, the electronic band structure of the GaAs(111)B surface before and after Bi incorporation was calculated by DFT, as shown in Figure 6d–f. Note that since our DFT calculation does not take into account the *GW* correction^27^ of the band gap, the local density approximation (LDA) used in the calculations underestimates the experimental band gap, as expected. This is because DFT eigenvalues are not quasiparticle excitation energies, and the *GW* approximation would properly include the electron self-energy effects. As a result, even though the experimental band gap of bulk zinc-blende GaAs is 1.51 eV at 10 K,^28^ the calculated DFT bulk band gap is 0.59 eV. Still, the calculations give a clear picture of the Bi-induced changes of the band structure. One may note that at a pristine (111)B surface, shown in Figure 6d, bands just above the band gap are mostly of Ga 4*s* character, while the states below the gap are mostly of As 4*p* character. The presence of unpassivated dangling bonds at the As-terminated (111)B surface results in *p*-type doping and trivial surface states at the Fermi level. Bi deposition leads to the formation of a honeycomb lattice with the two sublattices (Figure 5a). Even though some of the surface states persist at the Γ point after Bi deposition and incorporation (Figure 6f), two additional pairs of low-energy bands are formed just above and below the Fermi level in the low-energy regime that are of Bi 6*p*_*x*_ and 6*p*_*y*_ character.

To better understand the topological properties of the low-energy bands, we again solved the DFT Hamiltonian, but without contributions from the relativistic effects of spin–orbit coupling (SOC) (Figure 6e). In this model, partially occupied σ-bonds between the Bi 6*p*_*x*_ and 6*p*_*y*_ orbitals form two overlapping degenerate Dirac cones (i.e., four-fold degeneracy) at each of the two K points, with the Dirac points located at the Fermi level. The 8-fold degeneracy within the first Brillouin zone originates from the 2-fold degeneracies of each of the orbital, spin, and the sublattice degrees of freedom. The Bi 6*p*_*z*_ orbitals do not contribute to states at the Fermi level because they point out-of-the-plane (in the *z*-direction) and hybridize with the As orbitals below at the (111)B surface to form bonding and antibonding states above and below the Fermi level. Once SOC is included in the Hamiltonian (Figure 6f), the Bi 6*p*_*x*_ and 6*p*_*y*_ orbitals become coupled, leading to the opening of a nontrivial gap at the K points. Moreover, due to the hybridization of the Bi 6*p*_*z*_ orbitals with the substrate below the honeycomb monolayer, the 2-fold degeneracy due to the orbital degeneracy of *p*_*x*_ and *p*_*y*_ is no longer protected by inversion symmetry and is lifted through a Rashba-like perturbation. The nontrivial nature of the gap at the K points raises the exciting prospect of the Bi honeycomb lattice being used as a platform for exotic and nontrivial topological physics, e.g., as the host of quantum spin Hall phases or even fractional topological states under fractional fillings.^29^ LT-STS was performed to experimentally evaluate the projected band structure. Point spectra of the normalized differential conductance over varying voltage  were obtained on a clean GaAs(111)B surface and on the honeycomb structure resulting from Bi deposition and incorporation. Due to the high spatial stability of the LT-STM setup, it was possible to distinguish between spectra taken directly at a Bi atom and those taken at a hollow site of the honeycomb structure, as shown in Figure 6c. The normalized differential conductance is proportional to the LDOS of the surface^30^ and thus can give direct experimental information on surface band structure with atomic spatial resolution.^31^ The Fermi level is always at 0 V in STS results.

The spectrum obtained at the clean GaAs(111)B surface (black curve in Figure 6c) shows a large band gap without any states and a steep increase of the d*I*/d*V* signal at the VB and CB edges observed at about −2 V and +3 V, respectively. Thus, the apparent band gap amounts to 5 eV, which is much larger than the literature value of 1.51 eV. This is because of substantial tip-induced band bending,^22,32^ mainly due to the limited conductance of the GaAs substrate at low temperatures. In the spectra obtained at the honeycomb structure, both on (blue curve) and between (orange curve) the Bi atoms, the apparent band gap is significantly smaller: Here, the VB edge is observed as a steep increase of the states at about −1 V. For positive voltages, the CB can be recognized by a strong and steep increase of the d*I*/d*V* signal at voltages above 2 V. A linear extrapolation of this steep increase results in a CB edge at about 2 V. In addition, the d*I*/d*V* signal shows a strong peak at about 1.3 eV, which is within the band gap region. The intensity of this peak is larger for the spectra obtained at a hollow site compared to those obtained directly at a Bi atom. Some additional, weaker signal is obtained at energies slightly below the CB edge.

The significant shifts of the VB and CB edges to smaller absolute energies observed at the honeycomb structure, as compared to the clean GaAs(111)B surface, can indicate a Bi-induced decrease of the semiconductor band gap. However, it cannot be excluded that the presence of the surface honeycomb structure just limits the amount of tip-induced band bending. The absolute value of the band gap cannot finally be determined here. On the other hand, the peaks in the LDOS signal within the band gap would be affected by tip-induced band bending in the same way as the CB edge, thus their energy position relative to the CB edge can be considered as unaffected by the tip-induced band bending. Accordingly, the honeycomb structure is characterized by a strong Bi-induced state at about 0.7 eV below the CB edge and additional weaker states at about up to 0.2 eV below the CB edge. These results are in good qualitative agreement with the Bi-induced bands that by DFT calculations are shown to exist in the GaAs band gap.

The band inversion with a large gap opening at the K point, as resulting from the DFT model, is analogous to that of previously observed bismuthene formed on SiC^1^ or Ag(111).^4^ In the latter case as well as for (not yet experimentally realized) bismuthene on Si,^7^ DFT models showed large charge density between the Bi atoms and the Ag or Si surface atoms, indicating covalent bonds, similar to the structure studied here. This charge density was explained by strong hybridization of Bi *p*_*z*_ orbitals with dangling bonds of the surface atoms also in those studies. Furthermore, the authors attributed the relatively large nontrivial gap to the substrate-orbital-filtering effect, which due to the strong coupling between Bi and surface atom orbitals would move the Bi *p*_*z*_ bands away from the Fermi level. Such a strong interaction between the atoms of the honeycomb structure and those of the substrate underneath is uncommon for other Xene structures such as graphene or silicene and in contrast to very weak van der Waals interaction. However, it appears that both the strong spin–orbit coupling of Bi and the strong interaction with the substrate atoms are needed to obtain a large energy gap in these QSH systems. The GaAs:Bi honeycomb structure studied here, where XPS indicates covalent bonds only between the Bi and surface As atoms but not between neighboring Bi atoms, might thus be considered as an extreme case of a bismuthene system, giving promise for a large and robust nontrivial energy gap.

## Conclusion

In conclusion, our study demonstrates a Bi-induced 2D honeycomb structure on GaAs(111)B, revealing its atomic structure and illustrating a stable, well-ordered Bi terminated semiconductor surface with Bi-induced states within the GaAs band gap. The honeycomb structure is not limited to small individual flakes, but covers the entire GaAs(111)B substrate and follows its symmetry. Under optimized growth conditions, this honeycomb structure consists of Bi atoms which are exclusively bonded to As atoms, probably due to the relatively large distance between neighboring Bi atoms. The observed GaAs(111)B √3 × √3 R30° Bi structure corresponds to two-third of the surface As atoms being bonded to Bi atoms. Only upon additional Bi deposition or deposition at room temperature, the previously seen metallic Bi layer, characterized by Bi–Bi bonds, is forming on top. The successful Bi incorporation can be attributed both to the As-rich character of the GaAs(111) surface, which has a lower surface potential with Bi settled on the top of As atoms, and to the deposition at elevated temperature, providing the necessary activation energy for forming the honeycomb structure.

DFT calculations support the formation of the honeycomb structure and predict the presence of Bi-induced bands and the opening of a nontrivial gap at the K point. STS measurements of the projected band structure confirm the presence of Bi-induced states within the GaAs band gap. The Bi honeycomb structure observed here has a significantly larger lattice constant and a different bonding configuration than previously observed bismuthene structures. It seems to follow the same mechanism of becoming a 2D topological insulator, while the strong bonding between the Bi atoms and As atoms of the substrate, together with the strong spin–orbit coupling, is important for opening a large nontrivial gap. Furthermore, this structure forms directly and covalently bonded on GaAs. Thus, it enables the development of a next-generation design of devices, directly integrating spintronic applications of 2D topological insulators with well-established (opto)electronic semiconductor technology.

## Methods

### Sample Preparation

Commercial, *n-*doped GaAs(111)B wafers were rinsed by ethanol and introduced into ultrahigh vacuum (UHV, with a base pressure below 1 × 10^–10^ mbar) for STM imaging. The native oxide was removed by annealing the sample to 500 °C for around 1 h in a beam of atomic hydrogen, which has been proven to be a suitable way to clean III–V NWs.^22,33^ A thermal cracker from MBE Components was used for the cleaning procedure, operating at a cracking temperature of about 1700 °C and a hydrogen chamber pressure of 2 × 10^–6^ mbar. The Bi deposition was performed on the oxide-free sample, making use of a multichamber UHV cluster tool without breaking vacuum, using a resistant heating effusion cell from MBE components with a PBN crucible at a sample temperature of 250 °C. The temperature of the Bi cell was set to 400 °C–420 °C, corresponding to a low Bi flux, so that a deposition time of 30 min resulted in a deposited Bi amount of about 1 ML.

#### LT-STM/S

The readily prepared samples were analyzed in a low-temperature Sigma Infinity STM, operated in UHV at 10 K using a closed-cycle cryostat. Electrochemically etched tungsten tips that had been cleaned in vacuum by electron bombardment were used. Scanning was performed in constant current mode using sample bias, *V*_*T*_, and tunneling current, *I*_*T*_, as indicated. For STS measurements, the differential conductance d*I*/d*V* was recorded in parallel to the tunnel current using a lock-in-amplifier by modulating the DC bias voltage with an AC signal at a frequency of 1.0 kHz and a nominal peak-to-peak voltage of 40 mV. Several individually acquired STS spectra are averaged for noise reduction. The d*I*/d*V* signal was normalized by the broadened total conductance, as described in refs (32) and (33), and the resulting signal  represents the surface LDOS. For STS results obtained on a clean GaAs surface, a broadening constant of Δ*V* = 2.5 V was used, while, for STS on honeycomb structures, both on Bi atoms and on hollow sites, a broadening constant of Δ*V* = 2.0 V was used.

#### XPS

XPS measurements were obtained at MatLine beamline of the ASTRID2 Synchrotron facility at the University of Aarhus, Denmark. Ga *3d*, As *3d*, Bi *5d*, Bi *4f*, O *1s*, and C *1s* core-level spectra were obtained at varying photon energies. A polynomial background of fourth order was subtracted from the raw data. Spectra were fitted assuming a Voigt line profile with a spin–orbit splitting of 3.05 eV, a branching ratio of 0.8–0.85, and a Lorentzian width of 0.22 eV for Bi *5d* spectra. It should also be mentioned that the Bi *5d* core-level spectra with peak B.E. at 23.8 and 24.5 eV are partially overlapping with the strong Ga *3d* core level at about 19.5 eV, which can result in a slight discrepancy between raw and fitted data at the low binding-energy side of the Bi *5d* spectra.

#### DFT Calculations

The DFT calculations were performed for the Bi honeycomb structure as-deposited on a GaAs substrate. First, bulk GaAs of the zinc-blende crystal phase was constructed by relaxing its lattice parameter and the atomic positions using Quantum ESPRESSO,^34^ which uses a plane-wave basis set, such that all components of all forces were minimized within the convergence threshold of 10^–5^ Ry/bohr and the total energy was also minimized within the convergence threshold of 10^–8^ Ry. The plane-wave cutoff for the DFT calculation was set at 70 Ry for the plane-wave expansion of the wave functions using the scalar-relativistic GBRV ultrasoft pseudopotentials^35^ with nonlinear core correction.^36^ The LDA was used for the DFT exchange-correlation functional. The lattice parameter obtained was *a* = 0.561 nm, agreeing very well with the experimental literature values^37^ of *a* = 0.565 nm and our experiments (see Results and Discussion).

To model the pristine GaAs(111)B surface (i.e., As-terminated), a slab that was periodic in the **a**- and **b**-directions (Figure 5a) and a thickness of 1.5 unit cells (corresponding to three Ga–As planes) was constructed in a periodic supercell that is 2.5 nm long in the out-of-plane direction. Dangling bonds from Ga at the bottom of the slab were saturated using neutral pseudohydrogen atoms, each with a nuclear charge of 1.25*e* (where *e* is the positive elementary charge) to model the bulk of GaAs. To model the Bi honeycomb structure, two Bi atoms were placed above every three As atoms (i.e., at 67% coverage) at the As-terminated (111)B surface (at the top of the slab) at various configurations to model the experimental STM image (Figure 1). The atomic positions of all atoms constituting the pristine slab and the Bi-deposited slab were relaxed using the Vienna *ab initio* simulation program (VASP), except atoms in the third Ga–As plane from the top (Figure 5b), which were fixed in position to model bulk GaAs. The projector augmented wave (PAW) pseudopotentials within the LDA were used, and spin–orbit coupling was included. The structural optimization was performed using a Monkhorst–Pack **k**-grid of 6 × 6 × 1, a convergence threshold 10^–5^ eV/Å, and a 700 eV energy cutoff for the plane-wave basis set. Using the relaxed atomic structure, we calculated the DFT band structure as shown in Figure 6.

The formation energies (*E*_*f*_) needed to deposit Bi atoms in various configurations and the *E*_*f*_ needed to substitute the surface As atoms with Bi atoms were calculated. The formation energy of a particular configuration of atoms, *c*, in neutral state is defined as *E*_*f*_ = *E*_tot_^*c*^ – *E*_tot_^0^ + ∑_*i*_*n*_*i*_μ_*i*_ – ∑_*j*_*m*_*j*_μ_*j*_, where *E*_tot_^*c*^ is the total energy of the supercell in that configuration, *E*_tot_^0^ is the total energy of the corresponding pristine supercell, *n*_*i*_ is the number of atoms removed, *m*_*i*_ is the number of atoms added, and μ is the chemical potential of the atoms. The energy of Bi in the solid metal form, belonging to the space group of *R*3̅*m* and a rhombohedral lattice (*a* = 4.61 Å, *c* = 11.95 Å), is used as the chemical potential of Bi, μ_Bi_. The energy of As of the same crystal structure (*a* = 3.80 Å, *c* = 10.77 Å) is used as the chemical potential of As, μ_As_. The crystal lattices and atomic positions of bulk Bi and bulk As were both fully relaxed using VASP.

## Supporting Information Available

Detailed description and figures (Figure S1) of the clean GaAs(111)B surface after oxide removal with atomic hydrogen; LT-STM image of GaAs(111)B with a Bi-induced honeycomb structure (Figure S2), showing fluctuations of the electronic contrast. XPS results from As 3d and Ga 3d core-levels as well as the Valence Band (Figure S3). (PDF)

## References

[ref1] Reis F.; Li G.; Dudy L.; Bauernfeind M.; Glass S.; Hanke W.; Thomale R.; Schäfer J.; Claessen R. Bismuthene on a SiC substrate: A candidate for a high-temperature quantum spin Hall material. Science 2017, 357 (6348), 287–290. 10.1126/science.aai8142. 28663438

[ref2] Yao Y.; Ye F.; Qi X.-L.; Zhang S.-C.; Fang Z. Spin-orbit gap of graphene: First-principles calculations. Phys. Rev. B 2007, 75 (4), 041401 10.1103/PhysRevB.75.041401.

[ref3] Liu C.-C.; Feng W.; Yao Y. Quantum Spin Hall Effect in Silicene and Two-Dimensional Germanium. Phys. Rev. Lett. 2011, 107 (7), 076802 10.1103/PhysRevLett.107.076802. 21902414

[ref4] Sun S.; You J.-Y.; Duan S.; Gou J.; Luo Y. Z.; Lin W.; Lian X.; Jin T.; Liu J.; Huang Y. Epitaxial growth of ultraflat bismuthene with large topological band inversion enabled by substrate-orbital-filtering effect. ACS Nano 2022, 16 (1), 1436–1443. 10.1021/acsnano.1c09592. 34918901

[ref5] Yang F.; Elnabawy A. O.; Schimmenti R.; Song P.; Wang J.; Peng Z.; Yao S.; Deng R.; Song S.; Lin Y. Bismuthene for highly efficient carbon dioxide electroreduction reaction. Nat. Commun. 2020, 11 (1), 1–8. 10.1038/s41467-020-14914-9. 32107389 PMC7046785

[ref6] Liu X.; Zhang S.; Guo S.; Cai B.; Yang S. A.; Shan F.; Pumera M.; Zeng H. Advances of 2D bismuth in energy sciences. Chem. Soc. Rev. 2020, 49 (1), 263–285. 10.1039/C9CS00551J. 31825417

[ref7] Zhou M.; Ming W.; Liu Z.; Wang Z.; Li P.; Liu F. Epitaxial growth of large-gap quantum spin Hall insulator on semiconductor surface. Proc. Natl. Acad. Sci. U. S. A. 2014, 111 (40), 14378–14381. 10.1073/pnas.1409701111. 25246584 PMC4210051

[ref8] del Alamo J. A. Nanometre-scale electronics with III–V compound semiconductors. Nature 2011, 479 (7373), 317–323. 10.1038/nature10677. 22094691

[ref9] Nicolaï L.; Mariot J. M.; Djukic U.; Wang W.; Heckmann O.; Richter M. C.; Kanski J.; Leandersson M.; Balasubramanian T.; Sadowski J.; et al. Bi ultra-thin crystalline films on InAs(1 1 1)A and B substrates: a combined core-level and valence-band angle-resolved and dichroic photoemission study. New J. Phys. 2019, 21 (12), 123012 10.1088/1367-2630/ab5c14.

[ref10] Richter M.; Mariot J.-M.; Gafoor M.; Nicolaï L.; Heckmann O.; Djukic U.; Ndiaye W.; Vobornik I.; Fujii J.; Barrett N. Bi atoms mobility-driven circular domains at the Bi/InAs (111) interface. Surf. Sci. 2016, 651, 147–153. 10.1016/j.susc.2016.03.032.

[ref11] Ludeke R.; Taleb-Ibrahimi A.; Feenstra R. M.; McLean A. B. Structural and electronic properties of Bi/GaAs(110). Journal of Vacuum Science & Technology B: Microelectronics Processing and Phenomena 1989, 7 (4), 936–944. 10.1116/1.584584.

[ref12] Liu C.; Zhou Y.; Wang G.; Yin Y.; Li C.; Huang H.; Guan D.; Li Y.; Wang S.; Zheng H.; et al. Sierpiński Structure and Electronic Topology in Bi Thin Films on InSb(111)B Surfaces. Phys. Rev. Lett. 2021, 126 (17), 176102 10.1103/PhysRevLett.126.176102. 33988396

[ref13] McGinley C.; Cafolla A.; Murphy B.; Teehan D.; Moriarty P. The interaction of bismuth with the GaAs (111) B surface. Appl. Surf. Sci. 1999, 152 (3–4), 169–176. 10.1016/S0169-4332(99)00311-6.

[ref14] McGinley C.; Cafolla A. A.; McLoughlin E.; Murphy B.; Teehan D.; Moriarty P.; Woolf D. A. Core-level photoemission study of the Bi-GaAs(111)A interface. Appl. Surf. Sci. 2000, 158 (3), 292–300. 10.1016/S0169-4332(00)00012-X.

[ref15] Nakamura T.; Ohtsubo Y.; Yamashita Y.; Ideta S.-i.; Tanaka K.; Yaji K.; Harasawa A.; Shin S.; Komori F.; Yukawa R.; et al. Giant Rashba splitting of quasi-one-dimensional surface states on Bi/InAs(110)-(2 × 1). Phys. Rev. B 2018, 98 (7), 075431 10.1103/PhysRevB.98.075431.

[ref16] Honolka J.; Hogan C.; Vondráček M.; Polyak Y.; Arciprete F.; Placidi E. Electronic properties of GaAsBi (001) alloys at low Bi content. Physical Review Materials 2019, 3 (4), 044601 10.1103/PhysRevMaterials.3.044601.

[ref17] Cornille C.; Arnoult A.; Gravelier Q.; Fontaine C. Links between bismuth incorporation and surface reconstruction during GaAsBi growth probed by in situ measurements. J. Appl. Phys. 2019, 126 (9), 093106 10.1063/1.5111932.

[ref18] Laukkanen P.; Punkkinen M. P. J.; Lång J. J. K.; Sadowski J.; Kuzmin M.; Kokko K. Bismuth-containing c(4 × 4) surface structure of the GaAs(100) studied by synchrotron-radiation photoelectron spectroscopy and ab initio calculations. J. Electron Spectrosc. Relat. Phenom. 2014, 193, 34–38. 10.1016/j.elspec.2014.02.008.

[ref19] Duzik A.; Thomas J. C.; Van Der Ven A.; Millunchick J. M. Surface reconstruction stability and configurational disorder on Bi-terminated GaAs(001). Phys. Rev. B 2013, 87 (3), 035313 10.1103/PhysRevB.87.035313.

[ref20] Chuang F.-C.; Yao L.-Z.; Huang Z.-Q.; Liu Y.-T.; Hsu C.-H.; Das T.; Lin H.; Bansil A. Prediction of Large-Gap Two-Dimensional Topological Insulators Consisting of Bilayers of Group III Elements with Bi. Nano Lett. 2014, 14 (5), 2505–2508. 10.1021/nl500206u. 24734779

[ref21] Liu Y.; Knutsson J. V.; Wilson N.; Young E.; Lehmann S.; Dick K. A.; Palmstrøm C. J.; Mikkelsen A.; Timm R. Self-selective formation of ordered 1D and 2D GaBi structures on wurtzite GaAs nanowire surfaces. Nat. Commun. 2021, 12 (1), 5990 10.1038/s41467-021-26148-4. 34645829 PMC8514568

[ref22] Hjort M.; Lehmann S.; Knutsson J.; Timm R.; Jacobsson D.; Lundgren E.; Dick K. A.; Mikkelsen A. Direct Imaging of Atomic Scale Structure and Electronic Properties of GaAs Wurtzite and Zinc Blende Nanowire Surfaces. Nano Lett. 2013, 13 (9), 4492–4498. 10.1021/nl402424x. 23941328

[ref23] Bell G.; Kaijaks N.; Dixon R.; McConville C. F. Atomic hydrogen cleaning of polar III–V semiconductor surfaces. Surf. Sci. 1998, 401 (2), 125–137. 10.1016/S0039-6028(97)00914-X.

[ref24] Szamota-Leandersson K.; Leandersson M.; Göthelid M.; Karlsson U. O. Correlated development of a (2× 2) reconstruction and a charge accumulation layer on the InAs (111)–Bi surface. Surface science 2011, 605 (1–2), 12–17. 10.1016/j.susc.2010.09.015.

[ref25] Vondráček M.; Cornils L.; Minár J.; Warmuth J.; Michiardi M.; Piamonteze C.; Barreto L.; Miwa J. A.; Bianchi M.; Hofmann P.; et al. Nickel: The time-reversal symmetry conserving partner of iron on a chalcogenide topological insulator. Phys. Rev. B 2016, 94 (16), 161114 10.1103/PhysRevB.94.161114.

[ref26] Tersoff J.; Hamann D. R. Theory and application for the scanning tunneling microscope. Physical review letters 1983, 50 (25), 1998 10.1103/PhysRevLett.50.1998.

[ref27] Hybertsen M. S.; Louie S. G. Electron correlation in semiconductors and insulators: Band gaps and quasiparticle energies. Phys. Rev. B 1986, 34 (8), 5390 10.1103/PhysRevB.34.5390. 9940372

[ref28] http://www.ioffe.ru/SVA/NSM/Semicond/GaAs/basic.html.

[ref29] Claassen M.; Xian L.; Kennes D. M.; Rubio A. Ultra-strong spin–orbit coupling and topological moiré engineering in twisted ZrS_2_ bilayers. Nat. Commun. 2022, 13 (1), 4915 10.1038/s41467-022-31604-w. 35995779 PMC9395362

[ref30] Feenstra R. M. Tunneling spectroscopy of the (110) surface of direct-gap III-V semiconductors. Phys. Rev. B 1994, 50 (7), 4561–4570. 10.1103/PhysRevB.50.4561. 9976760

[ref31] Knutsson J. V.; Lehmann S.; Hjort M.; Lundgren E.; Dick K. A.; Timm R.; Mikkelsen A. Electronic Structure Changes Due to Crystal Phase Switching at the Atomic Scale Limit. ACS Nano 2017, 11, 10519–10528. 10.1021/acsnano.7b05873. 28960985

[ref32] Feenstra R. M.; Stroscio J. A. Tunneling spectroscopy of the GaAs (110) surface. Journal of Vacuum Science & Technology B: Microelectronics Processing and Phenomena 1987, 5 (4), 923–929. 10.1116/1.583691.

[ref33] Knutsson J.; Lehmann S.; Hjort M.; Reinke P.; Lundgren E.; Dick K.; Timm R.; Mikkelsen A. Atomic scale surface structure and morphology of InAs nanowire crystal superlattices: the effect of epitaxial overgrowth. ACS Appl. Mater. Interfaces 2015, 7 (10), 5748–5755. 10.1021/am507931z. 25710727 PMC4382987

[ref34] Giannozzi P.; Baroni S.; Bonini N.; Calandra M.; Car R.; Cavazzoni C.; Ceresoli D.; Chiarotti G. L.; Cococcioni M.; Dabo I.; et al. QUANTUM ESPRESSO: a modular and open-source software project for quantum simulations of materials. J. Phys.: Condens. Matter 2009, 21 (39), 395502 10.1088/0953-8984/21/39/395502. 21832390

[ref35] Vanderbilt D. Soft self-consistent pseudopotentials in a generalized eigenvalue formalism. Phys. Rev. B 1990, 41 (11), 7892 10.1103/PhysRevB.41.7892. 9993096

[ref36] Louie S. G.; Froyen S.; Cohen M. L. Nonlinear ionic pseudopotentials in spin-density-functional calculations. Phys. Rev. B 1982, 26 (4), 1738 10.1103/PhysRevB.26.1738.

[ref37] Blakemore J. Semiconducting and other major properties of gallium arsenide. J. Appl. Phys. 1982, 53 (10), R123–R181. 10.1063/1.331665.

