# Supplementary material for: A 2D Bismuth-Induced Honeycomb Surface Structure on GaAs(111)
Source: ACS Nano. 2023 Feb 23;17(5):5047–58. doi: 10.1021/acsnano.2c12863 (PMC10018767; doi:10.1021/acsnano.2c12863)
Supplement: Supplementary file 1 — nn2c12863_si_001.pdf [file nn2c12863_si_001.pdf]

Supplementary Information for:

A 2D bismuth-induced honeycomb surface structure on GaAs(111)

Yi Liu[1], Sandra Benter[1], Chin Shen Ong[2], Renan P. Maciel[2], Linnéa Björk[1], Austin Irish[1], Olle Eriksson[2,3], Anders Mikkelsen[1], and Rainer Timm[1]

[1]NanoLund and Department of Physics, Lund University, P.O. Box 118, 221 00 Lund, Sweden

[2]Department of Physics and Astronomy, Uppsala University, P.O. Box 516, 751 20 Uppsala, Sweden

[3]School of Science and Technology, Örebro University, Fakultetsgatan 1, SE-70182 Örebro, Sweden

Content:

Clean GaAs(111)B surface after oxide removal with atomic hydrogen page 2

Fig. S1: LT-STM images of oxide-free GaAs(111)B substrate at around 10K page 2

Fig. S2: LT-STM image of GaAs(111)B with a Bi-induced honeycomb structure, showing fluctuations of the electronic contrast page 3

XPS results from As 3d and Ga 3d core-levels as well as the Valence Band page 4

Fig. S3: Complementary XPS results page 4

Supplementary References page 4

Clean GaAs(111)B surface after oxide removal with atomic hydrogen. The thin oxide layer on the surface of the GaAs(111)B substrate, resulting from air exposure, was removed by annealing the sample at around 500°C under a flux of atomic hydrogen. LT- STM images of the resulting oxide-free GaAs(111)B surface, terminated by As atoms, are shown in Fig. S1. Triangular shaped terraces with terrace edges along <110> directions can be clearly seen; this is a strong evidence of an oxide-free (111) surface[1].

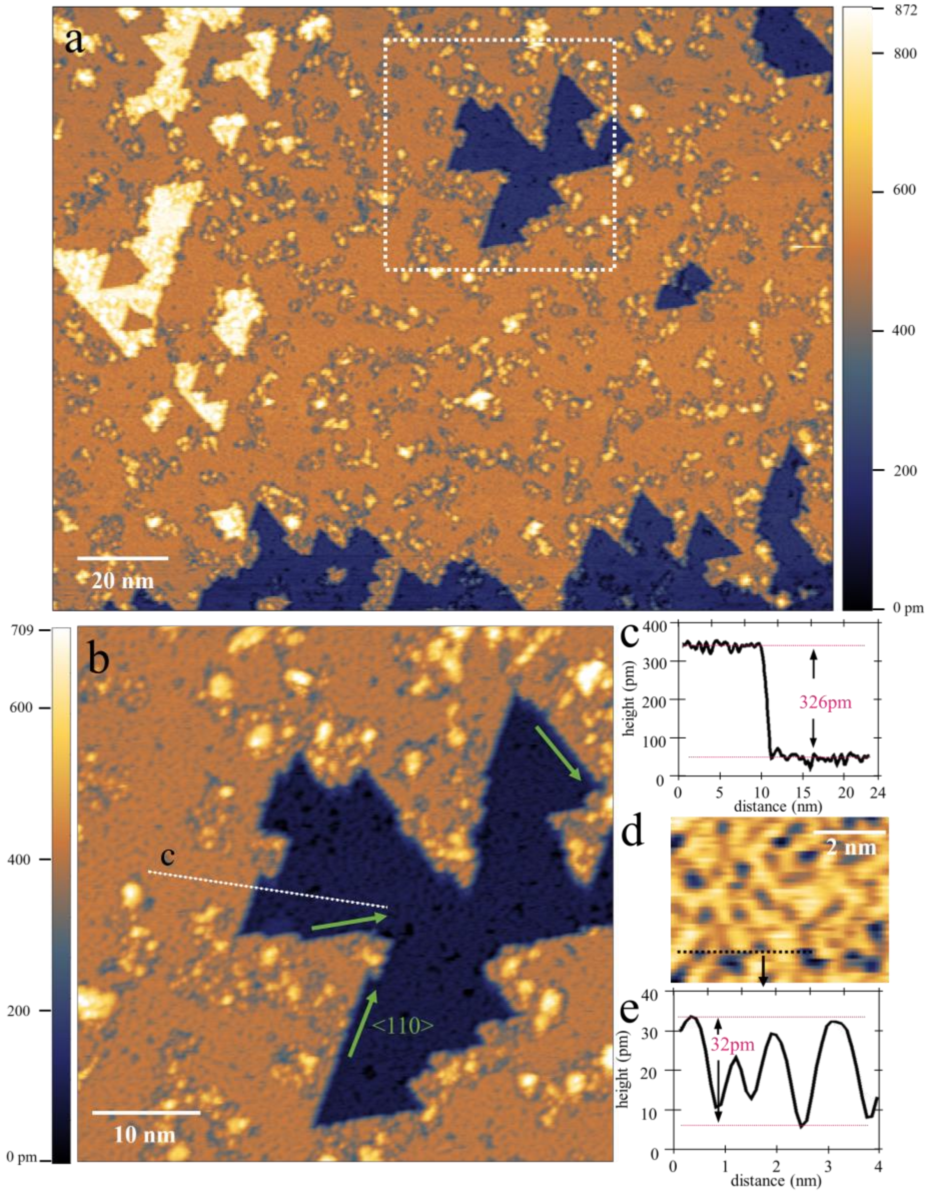

Figure S1: LT-STM images of oxide-free GaAs(111)B substrate at around 10K. (a) Overview STM image. (b) Zoom-in STM image of the rectangular area marked by white dashed lines in (a). The green arrows indicate terrace edges along <110> directions. (c) Height profile crossing a terrace edge, as indicated in (b) by the white dashed line (b). (d) Atomic-scale STM image, showing an irregular nm-scale contrast pattern. (e) Height profile of the irregular pattern along the black dashed line marked in (d).

Interestingly, on the clean GaAs(111) surface, unordered nm-scale fluctuations of the local density of states (LDOS) are observed in the LT-STM images, as shown in Fig. S1d, with the height profile along the black dashed line shown in Fig. 1e. The apparent topography variation of about 20 pm can principally be resulting both from electronic contrast changes due to variations of the local density of states (LDOS) or from surface

topography. Oxide free GaAs(111)B measured with STM at room temperature show no such irregular patterns, instead, an ordered crystalline (111) structure is observed on the surface[1]. The irregular pattern, only observed at low temperature, might indicate the presence of a condensed 2D electron gas (2DEG) at 10 K[2,3]. However, similar irregular fluctuations of the contrast are even observed after formation of the Bi-induced honeycomb network, as shown in Fig. S2 and in Fig. 4 of the main manuscript. The formation of the honeycomb structure should prohibit the presence of a surface 2DEG. Thus, we rather attribute the irregular contrast fluctuations to nm-scale changes of the LDOS at the GaAs surface, probably due to dopants and/or defects in the sub-surface layers.

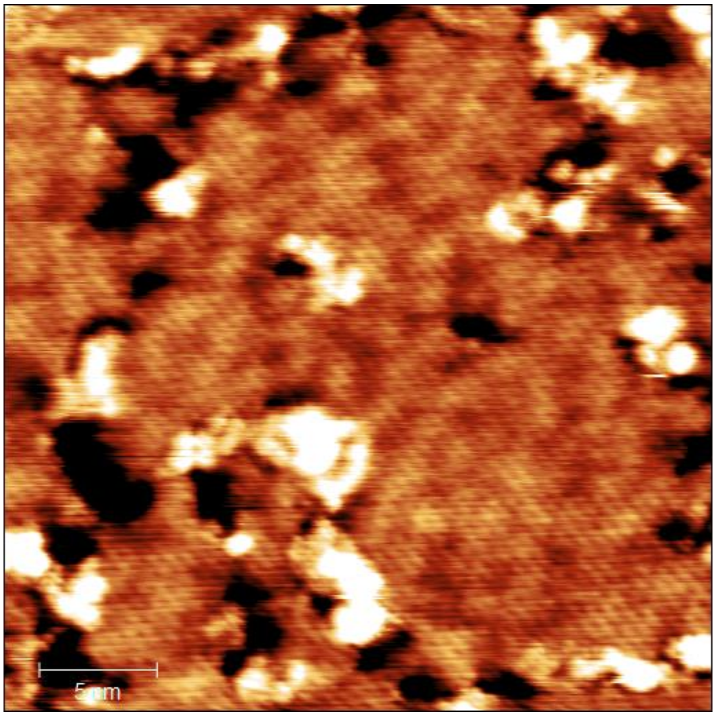

Figure S2: LT-STM image of GaAs(111)B with a Bi-induced honeycomb structure, showing fluctuations of the electronic contrast of the GaAs substrate in addition to the regular honeycomb network.

XPS results from As 3d and Ga 3d core-levels as well as the Valence Band. XPS results from the Bi *5d* core level are presented in Fig. 3 of the main manuscript. Complementary results from As *3d* and Ga *3d* core levels as well as the valence band are shown here in Fig. S3.

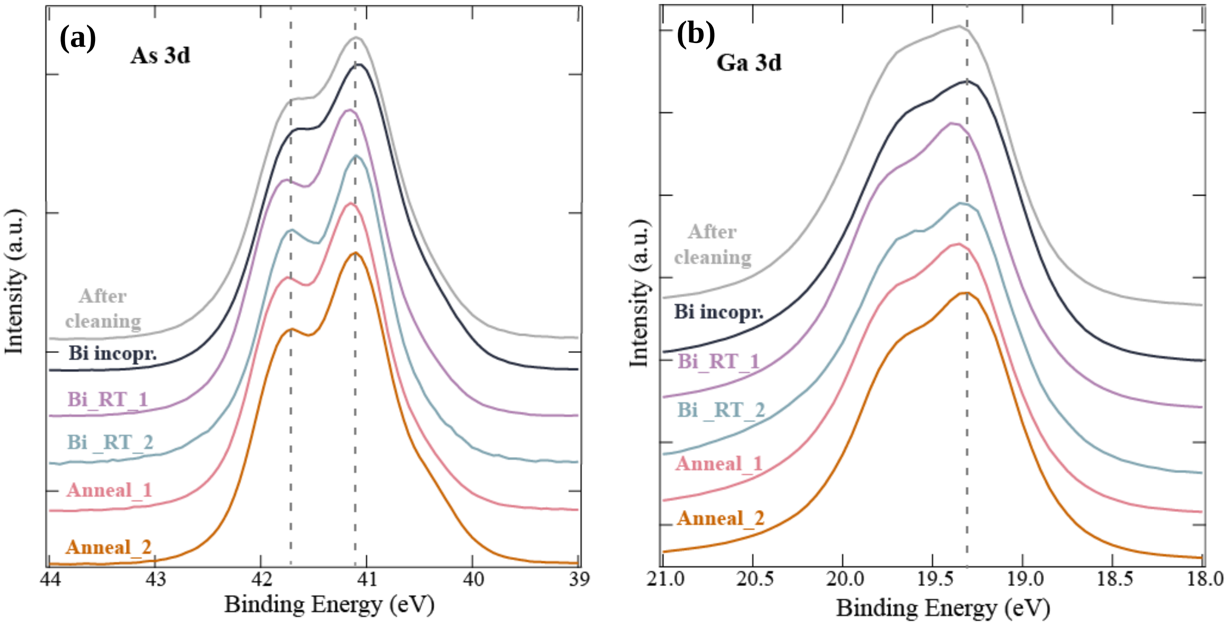

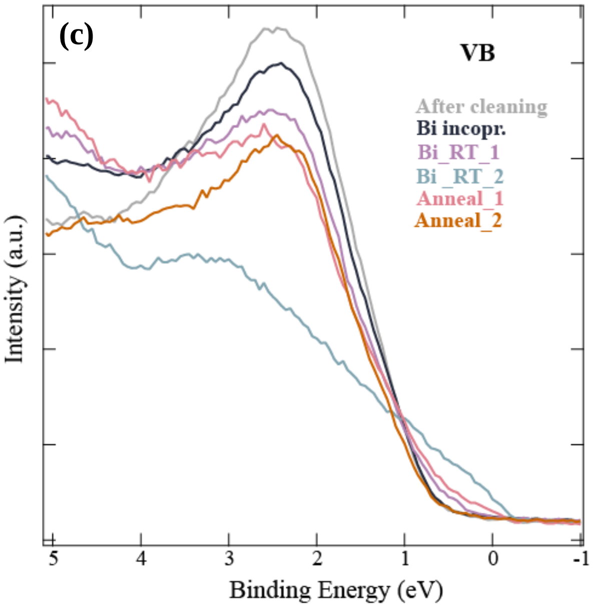

Figure S3: Complementary XPS results. (a) As *3d*, (b) Ga *3d*, and (c) valence band spectra after subsequent processing steps, as described in the main text. The B.E. positions of the As *3d* 5/2 and 3/2 peaks and of the Ga *3d* 5/2 peak from the clean sample are indicated by dashed lines, respectively.

References:

1 Hilner, E., Lundgren, E. & Mikkelsen, A. Surface structure and morphology of InAs(111)B with/without gold nanoparticles annealed under arsenic or atomic hydrogen flux. *Surface Science* **604**, 354-360, doi:https://doi.org/10.1016/j.susc.2009.11.029 (2010).

2 Morgenstern, M., Klijn, J., Meyer, C., Römer, R. A. & Wiesendanger, R. Comparing measured and calculated local density of states in a disordered two-dimensional electron system. *Physica B: Condensed Matter* **329-333**, 1536-1537, doi:https://doi.org/10.1016/S0921-4526(02)02279-2 (2003).

3 Wenderoth, M. *et al.* Low-temperature scanning tunneling spectroscopy as a probe for a confined electron gas. *Europhysics Letters (EPL)* **45**, 579-584, doi:10.1209/epl/i1999-00206-0 (1999).
